# Effect of Different Climate Change Variables on the Ecology and Management of *Sesbania cannabina* through Glyphosate

**DOI:** 10.3390/plants10050910

**Published:** 2021-05-01

**Authors:** Nadeem Iqbal, Sudheesh Manalil, Bhagirath Singh Chauhan, Steve Adkins

**Affiliations:** 1 School of Agriculture and Food Sciences, The University of Queensland, Gatton, QLD 4343, Australia; b.chauhan@uq.edu.au (B.S.C.); s.adkins@uq.edu.au (S.A.); 2 The Centre for Crop Science, Queensland Alliance for Agriculture and Food Innovation (QAAFI), The University of Queensland, Gatton, QLD 4343, Australia; s.manalil@uq.edu.au; 3 School of Agriculture and Environment, Institute of Agriculture, The University of Western Australia, Crawley, Perth, WA 6009, Australia; 4 Amrita School of Agricultural Sciences, Amrita Vishwa Vidyapeetham, Coimbatore 641112, India

**Keywords:** elevated [CO_2_], ambient [CO_2_], moisture stress, *S. cannabina*, glyphosate

## Abstract

An elevated atmospheric carbon dioxide (CO_2_) concentration and frequent droughts are two anticipated climate change scenarios in which certain invasive weeds may develop competitive advantages over crops and adversely impact productivity and herbicide efficacy. Hence, a study was conducted to explore the effect of different climatic scenarios on the growth and management of *Sesbania cannabina* (Retz.) Pers with glyphosate. The variables investigated were two CO_2_ concentrations (400 and 700 ppm), two soil moisture levels (100% and 50% of field capacity (FC)), and three glyphosate rates (0 (control), 517 (50% of recommended rate), and 1034 g ae ha^−1^ (recommended rate)). CO_2_ concentrations and soil moisture levels had different effects on the growth and management of *S. cannabina*. Overall, 100% FC and elevated [CO_2_] of 700 ppm recorded the maximum plant height (38 cm), leaves per plant (20), growth index (60), chlorophyll content (SPAD value 37), and dry biomass (3 g) in comparison with ambient [CO_2_] of 400 ppm and 50% FC treatment. The recommended glyphosate application gave 100% weed biomass reduction; however, efficacy was reduced (63%) when applied at 50% of the recommended rate under elevated [CO_2_] of 700 ppm and 50% FC conditions.

## 1. Introduction

The changes in global climate resulting from emissions of greenhouse gases are impacting both agricultural and natural ecosystems, reducing native biodiversity, agricultural productivity, and global food security [1,2]. Currently, the key climatic variables identified as important to plant growth are rising atmospheric [CO_2_], warming temperatures, and uneven rainfall patterns, leading to droughts and floods [3]. Amongst these changes, the rising atmospheric [CO_2_] and drought are the two variables having the most immediate impact on the physiology and reproductivity of plants, including weeds [3]. The atmospheric [CO_2_] in the pre-industrial era was 280 ppm, which has now increased to >400 ppm, mainly due to enhanced industrial activity brought about by a rising human population [4]. According to the latest predictions, the atmospheric [CO_2_] may surpass 550 ppm by 2050 and 700 ppm by 2100 [2]. This rising atmospheric [CO_2_] can influence the growth and productivity of certain crop plants and weeds through adjusting their physiological, biochemical, and reproductive processes, with the impact being more conspicuous for C3 plants, with an increase in their photosynthetic activity by up to 200% [5,6,7,8].

The changing climate caused by the elevated atmospheric [CO_2_] is also causing an increase in global air temperatures, causing a greater number of heat waves and drought events [9,10]. This reduction in soil moisture levels may reduce plant growth, development, and reproduction of both crops and weeds [11,12]. However, many weed species, such as *Hordeum murinum* L., *Bromus tectorum* L., and *Lactuca serriola* L., are better adapted to these climatic conditions due to their phenotypic and biological plasticity [13]. Well-adapted weed species in a changing climate scenario (elevated [CO_2_] and drought conditions) will become a bigger challenge in crop production [3]. Hence, proper weed management is indispensable for maximizing crop productivity by involving all control strategies, including “biological, cultural, mechanical and chemical” [14,15,16].

Among the herbicides, glyphosate has become one of the most successful herbicides due to its effectiveness against most types of dicotyledonous and monocotyledonous weeds, including *S. cannabina*. Glyphosate inhibits the action of 5-enolpyruvylshikimate-3-phosphate synthase (EPSPS), an enzyme involved in the production of aromatic amino acids by the shikimate pathway [14,15,16,17]. Glyphosate enters the plants through the foliage and is translocated via the phloem to the sink tissues [5]. Due to this, the efficacy of glyphosate can be affected by several environmental factors, including soil moisture stress, ambient temperature and relative humidity, and light intensity [5,9]. There are also reports from previous studies that elevated [CO_2_] and soil moisture stress can reduce the efficacy of glyphosate [18,19,20]. For example, the efficacy of glyphosate was reduced when *Elytrigia repens* (L.) Desv. Ex Nevski. and *Cirsium arvense* (L.) Scop. were treated in an elevated [CO_2_] environment [11,20]. However, other studies have reported that elevated [CO_2_] does not impact glyphosate efficacy [13,21]. In a recent study, it was reported that elevated [CO_2_] in rising temperature levels could affect the growth parameters of various weeds but does not have any impact on glyphosate efficacy [13].

*Sesbania cannabina* (Retz.) Pers, a C3 annual Fabaceae shrub, is rapidly becoming a ubiquitous problematic weed in the dryland and irrigated cotton production regions of Australia [22,23,24]. It not only impacts cotton production, but heavy infestations also reduce lint yield substantially and deteriorate its quality, as well as interfering with harvesting operations [25]. *Sesbania cannabina* can grow well in heavy loam soils, and plants can withstand saline, waterlogged, and alkaline conditions [22]. In Australia, successive cohorts can emerge in spring, summer, and autumn, with summer conditions being ideal for rapid growth and development and late spring to early autumn being ideal for flowering and seed production [22]. A single plant can produce up to 1200 pods, which can result in the production of 24,000–42,000 seeds [22]. Being a C3 plant, changing climatic conditions such as elevated [CO_2_] and drought may affect its growth and management. No information is yet available in the literature regarding the biology and management of *S. cannabina* in a changing climate.

Considering these observations and the growing problem of *S. cannabina* in Australian cotton production, a study was conducted with the following research objectives:

- To evaluate whether different atmospheric [CO_2_] and soil moisture levels can affect the growth of *S. cannabina* through changes in its morphological and physiological parameters
- To determine the interactive impact of elevated [CO_2_] and moisture stress conditions on the efficacy of glyphosate

## 2. Results

### 2.1. Impact of Atmospheric [CO_2_] and Soil Moisture Levels on Growth

**Figure 1:**
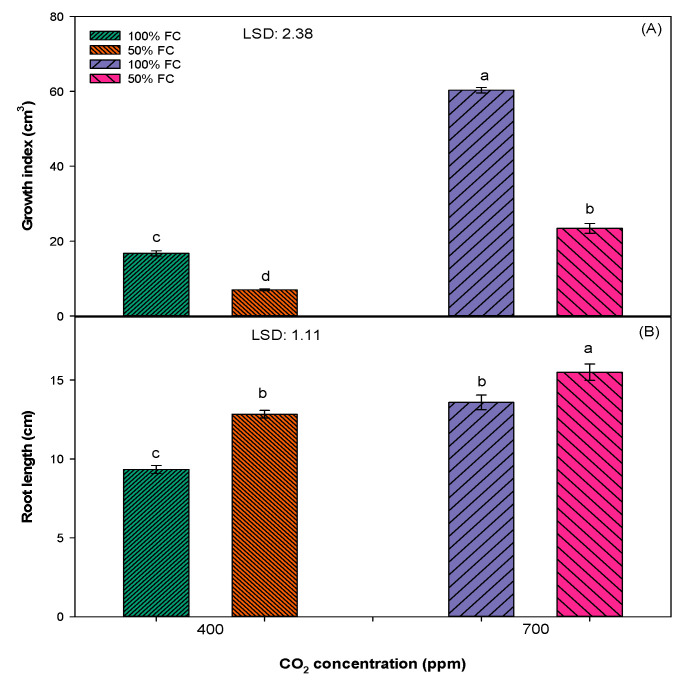
Effect of different [CO_2_] and soil moisture levels on (**A**) plant height and (**B**) leaves per plant of *S. cannabina* recorded at harvest. The experiment was repeated, and the pooled data from the two studies are presented here. The vertical bars represent error bars (*n* = 12). LSD represents the least significance difference (*p* ≤ 0.05; *n* = 12). Different letters (a, b, c, d) on the error bars indicate significant differences, as determined by Fisher’s protected LSD test at *p* < 0.05. The abbreviation FC in the figure represents the field capacity.

**Figure 2:**
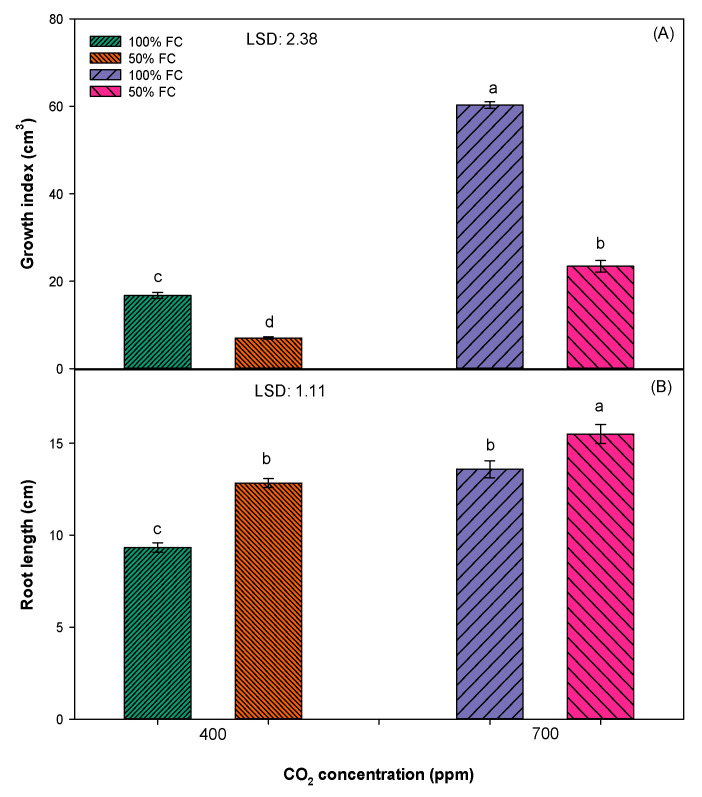
Effect of different [CO_2_] and soil moisture levels on (**A**) growth index and (**B**) root length of *S. cannabina*. The experiment was repeated, and the pooled data from the two studies are presented here. The vertical bars represent error bars (*n* = 12). LSD represents the least significance difference (*p* ≤ 0.05; *n* = 12). Different letters (a, b, c, d) on the error bars indicate significant differences, as determined by Fisher’s protected LSD test at *p* < 0.05. The abbreviation FC in the figure represents the field capacity.

Overall, the increase in all parameters (viz., plant height, leaves per plant, and growth index) were greatest in an elevated [CO_2_] of 700 pm as compared to an ambient [CO_2_] of 400 ppm. The maximum plant height (38 cm), leaves per plant (20), and growth index (60) of *S. cannabina* were recorded in 100% field capacity (FC) treatment and in the elevated [CO_2_] of 700 ppm, except for the root length, which was higher (16 cm) in 50% FC and an elevated [CO_2_] of 700 ppm (Figure 1 and Figure 2). Soil water stress negatively affected the growth of *S. cannabina*; however, this effect was most severe in the ambient [CO_2_] of 400 ppm, and plants grown under these conditions were shorter in stature (11 cm), had fewer leaves per plant (7), and a low growth index (7) in comparison with plants grown in 50% FC and an elevated [CO_2_] of 700 ppm treatment (Figure 1 and Figure 2).

**Figure 3:**
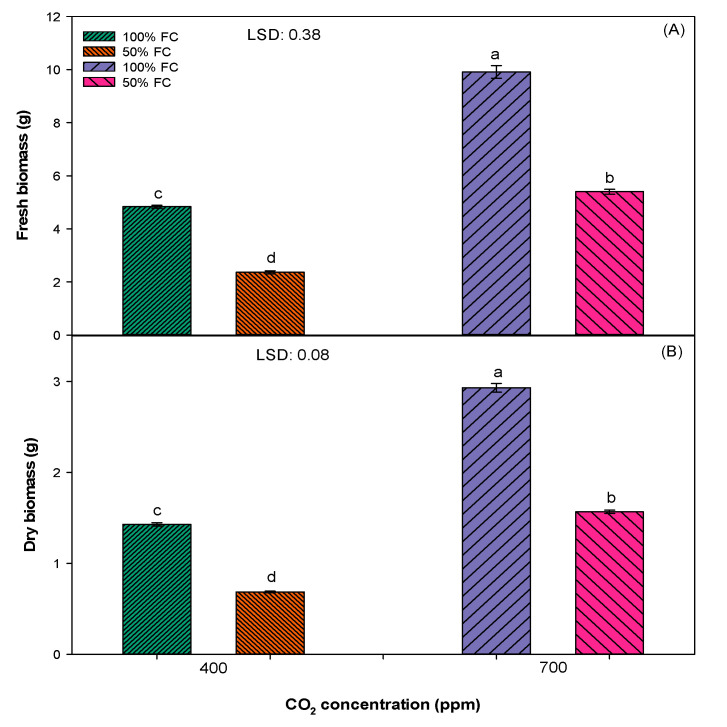
Effect of different [CO_2_] and soil moisture levels on (**A**) fresh biomass and (**B**) dry biomass of *S. cannabina*. The experiment was repeated, and the pooled data from the two studies are presented here. The vertical bars represent error bars (*n* = 12). LSD represents the least significance difference (*p* ≤ 0.05; *n* = 12). Different letters (a, b, c, d) on the error bars indicate significant differences, as determined by Fisher’s protected LSD test at *p* < 0.05. The abbreviation FC in the figure represents the field capacity.

*S. cannabina* produced greater fresh (10 g) and dry (3 g) biomass when grown in 100% FC and an elevated [CO_2_] of 700 ppm as compared to plants grown in 100% FC and an ambient [CO_2_] of 400 ppm or 50% FC and an ambient [CO_2_] of 400 ppm (Figure 3). The lowest fresh (2 g) and dry (1 g) biomass was recorded in 50% FC and an ambient [CO_2_] of 400 ppm.

**Figure 4:**
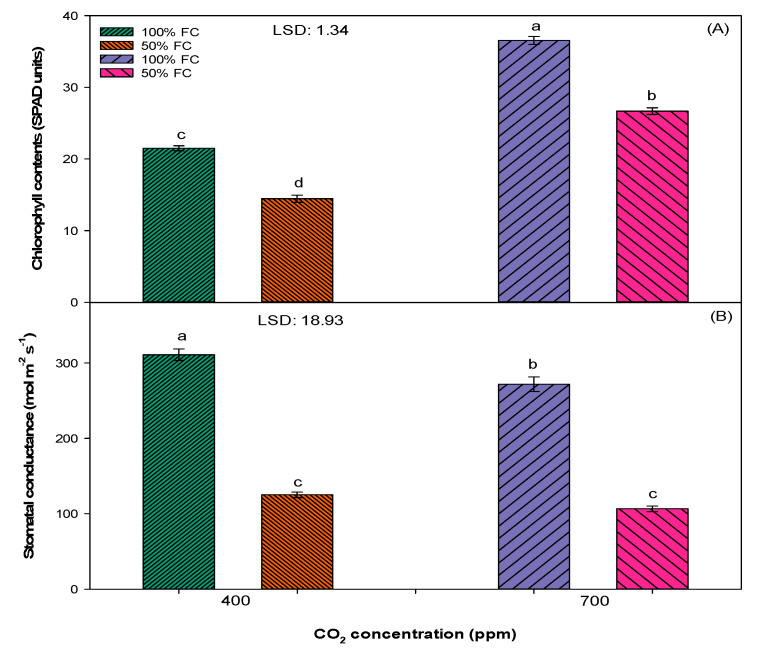
Effect of different [CO_2_] and soil moisture levels on (**A**) chlorophyll content (SPAD value) and (**B**) stomatal conductance of *S. cannabina*. The experiment was repeated, and the pooled data from the two studies are presented here. The plotted data represent the combined data set of two experiments. The vertical bars represent error bars (*n* = 12). LSD represents the least significance difference (*p* ≤ 0.05; *n* = 12). Different letters (a, b, c, d) on the error bars indicate significant differences, as determined by Fisher’s protected LSD test at *p* < 0.05. The abbreviation FC in the figure represents the field capacity.

The different [CO_2_] and soil moisture levels also affected the chlorophyll content and stomatal conductance (Figure 4). Plants grown in 100% FC and an elevated [CO_2_] of 700ppm recorded the highest chlorophyll content (SPAD value 37). This was followed by plants grown in 50% FC and an elevated [CO_2_] of 400 ppm (SPAD value 27) and in 100% FC and an ambient [CO_2_] of 400 ppm (SPAD value 22). The lowest chlorophyll content (SPAD value 15) was recorded in 50% FC and an ambient [CO_2_] of 400 ppm. Plants grown in an elevated [CO_2_] of 700 ppm exhibited lower stomatal conductance (218 mmol s^−1^ m^−1^) as compared to plants grown in an ambient [CO_2_] of 400 ppm (189 mmol s^−1^ m^−1^). The lower stomatal conductance in the elevated [CO_2_] of 700 ppm may be due to improved water use efficiency (WUE). Comparing the effect of different soil moisture levels, plants grown in 100% FC treatment recorded higher stomatal conductance (291 mmol s^−1^ m^−1^) than plants grown in 50% FC treatment (116 mmol s^−1^ m^−1^) (Figure 4).

### 2.2. Impact of Different [CO_2_] and Soil Moisture Levels on Glyphosate Efficacy

Results revealed that glyphosate application at the recommended rate (1034 g ae ha^−1^) resulted in higher mortality and maximum reduction in the weed biomass of *S. cannabina* under all climatic conditions studied. However, when glyphosate application was reduced to 50% of the recommended rate (517 g ae ha^−1^), 50% FC and an elevated [CO_2_] of 700 ppm treatment reduced the efficacy of glyphosate and resulted in a lower biomass reduction (63%) in *S. cannabina*, followed by 50% FC and an ambient [CO_2_] of 400 ppm treatment (94%; Table 1). Similar observations were recorded on the weed control rating scale. The 50% FC and an elevated [CO_2_] of 700 ppm recorded only a 46% weed control rating of glyphosate, followed by 50% FC and an ambient [CO_2_] of 400 ppm (87%; Table 2).

## 3. Discussion

### 3.1. Effect of Different [CO_2_]

The present research suggests that *S. cannabina* grown in an elevated [CO_2_] of 700 ppm will be taller, with more leaves, a high growth index, longer root length, greater fresh and dry biomass, and greater chlorophyll content when compared to plants grown in an ambient [CO_2_] of 400 ppm (Figure 1, Figure 2, Figure 3 and Figure 4). These findings acquiesce with others in predicting that an elevated [CO_2_] of 700 ppm will have a significant positive effect on the growth and biomass production of *S. cannabina* [13,26]. In a previous study, *Xanthium strumarium* L., a C3 weed, when grown in an elevated [CO_2_] environment presented a higher leaf area, leaf photosynthesis, and dry biomass [27]. A similar trend was observed in several other C3 broadleaf weeds such as *Abutilon theophrasti* Medik., *Chenopodium album* L., *Datura stramonium* L., *Cirsium arvense* L., and *Parthenium hysterophorus* L. [28,29].

The main reasons for the higher growth and development of *S. cannabina* seen in an elevated atmospheric [CO_2_] is thought to be related to the greater photosynthetic rate brought about by C3 photosynthesis and a lower photorespiration rate [30,31]. Another response for the higher productivity of *S. cannabina* in the elevated atmospheric [CO_2_] may be the reduced stomatal conductance (Figure 4). As a result, water loss from the plant is minimized due to partial closure of stomata, while CO_2_ uptake is maintained at a higher rate [6,8]. Hence, plants grown in an elevated [CO_2_] are at more advantage as compared to plants grown in an ambient [CO_2_], and this can result in improved growth and biomass production, as observed in this study [30,31].

### 3.2. Effect of Different Soil Moisture Levels

Results revealed that soil moisture stress has a negative effect on the growth and development of *S. cannabina* in either an elevated [CO_2_] of 700 ppm or an ambient [CO_2_] of 400 ppm (Figure 1, Figure 2, Figure 3 and Figure 4). Plants grown in 50% FC treatment recorded less growth and biomass production, except the root length, in comparison to plants grown in 100% FC treatment. These results concur with previous studies in glasshouses where negative effects of soil moisture stress in terms of both intensity and frequency on the growth and reproduction of *S. cannabina* were observed (unpublished data). A negative effect of moisture stress on growth and biomass production has also been reported in some other broadleaf weeds, such as *Parthenium hysterophorus, Amaranthus rudis L.*, and *Ambrosia trifida L.*, in previous studies [32,33,34].

Moisture stress inversely affects the photosynthetic efficiency of plants by decreasing the supply of CO_2_ through the closure of stomata, decreasing the activity of ribulose-1,5-bisphosphate carboxylase/oxygenase (RuBisCo), and increasing the activity of photorespiration (a waste process in which plants use substrate and energy) [30]. Unlike other growth parameters, the root length increased under moisture stress conditions (Figure 3). Similar to the results of this study, a positive effect of moisture stress on root length was reported in other study, where the root length of *P. hysterophorus* increased with an increase in the water stress intensity (from 100% to 50% FC) [32]. However, a reverse trend was observed in the root length of *A. rudis*, which decreased with an increase in the intensity of water stress (from 100% to 12.5% FC) [34]. The ability of plants to elongate roots under moisture stress conditions is an adaptation to thrive in drought [35].

Different from the ambient [CO_2_], the effect of moisture stress was less severe in plants grown in an elevated atmospheric [CO_2_] (Figure 1, Figure 2, Figure 3 and Figure 4). Plants grown in an elevated [CO_2_] have high water use efficiency (WUE) due to lower stomatal conductance and lower water loss through transpiration (Figure 4) [36]. In a previous study, it was reported that the WUE of the C3 weed *P. hysterophorus* increased when plants were grown in an elevated atmospheric [CO_2_] of 550 ppm as compared to an ambient [CO_2_] of 380 ppm [29]. The results from the present study showed enhanced growth of *S. cannabina* in an elevated atmospheric [CO_2_] and with moisture-limiting treatment. This has an important implication in the future climate regime, such as further spread and infestation of *S. cannabina* in the dryland cropping regions of Australia.

### 3.3. Efficacy of Glyphosate in Climate Change

Irrespective of different treatments, glyphosate at the recommended rate (1034 g ae ha^−1^) resulted in maximum biomass reduction of *Sesbania cannabina* (Table 1 and Table 2). These results concur with a previous study where glyphosate application at 1440 g ae ha^−1^ (recommended rate) resulted in 100% biomass reduction in *B. tectorum*, *H. murinum*, and *L. serriola* in either an ambient or an elevated [CO_2_] [13]. However, these results are contrary to some of the previous findings where elevated [CO_2_] and drought conditions altered the activity of glyphosate [15,37]. This may be the due to the different genetic makeup of those weed species that are well adapted to the climate change scenario and show tolerance when exposed to elevated atmospheric [CO_2_] and moisture stress conditions [38,39]. For example, *Conyza canadensis* (L.) Cronq. ERICA and *Chenopodium album* L. showed reduced sensitivity to glyphosate when grown under elevated [CO_2_] and increased temperature conditions [15].

It was also found in this study that the activity of glyphosate reduced in 50% FC and an elevated [CO_2_] of 700 ppm when applied at a lower rate of 517 g ae ha^−1^ (50% of the recommended rate). Cutting the herbicide rate below the standard recommendation is a poor application of agrochemicals, which can adversely affect the herbicide efficiency, as in this study, and can lead to herbicide resistance [40,41]. These results concur with a previous study where *Lolium rigidum* Guad (rigid ryegrass) survived a lower dose of diclofop-methyl herbicide and later developed resistance in both pot and field experiments [41]. Moisture stress is another key factor linked with the reduced efficiency of glyphosate [18]. Some previous studies reported the lowest efficacy of glyphosate for the management of *A. fatua* and *U. panicoides* when spraying was done under water stress conditions of 29% FC [42]. Furthermore, moisture stress also reduced the absorption and translocation of glyphosate, which may reduce its activity in plants, as in this study [7]. An elevated [CO_2_] also helped *S. cannnbina* to show less susceptibility to glyphosate when application was done at a reduced rate [3]. This may be due to the increased growth of this weed in an elevated atmospheric [CO_2_] as compared to an ambient [CO_2_] [3]. These results are relevant as there are operational (less delivery through a faulty nozzle) and environmental factors, and intentional usage of low herbicide dosages that may result in a reduction in the herbicide active ingredient being delivered on weeds, and few escaping weeds with abundant seed production can lead to heavy weed infestation and herbicide resistance such as in *L. rigidum* [40,41]. Hence, herbicides should always be applied as per label recommendations.

## 4. Materials and Methods

### 4.1. Seed Collection, Storage, and Planting

Seeds of *S. cannabina* were collected from a cropping field in a moderately high rainfall region of Dalby, Queensland (540 mm per annum; 27° 22.011 S, 151° 15.674 E), on 07-04-2016. Seeds were stored after harvest under laboratory conditions prior to being used. At harvest and after 6 months of storage, seeds exhibited physical dormancy. To overcome this physical dormancy, seeds were subjected to hot-water treatment (100 ± 2 °C) for 5 min in a Gallenkemp water bath [23]. After the hot-water treatment, the seeds were blotted dry and then sown at a depth of 2 cm in 72 free-draining black plastic pots (20 × 20 cm^2^, diameter by height) containing finely ground soil collected from the Experimental Research Farm, University of Queensland, Gatton, Australia.

The soil was heavy clay loam having an electrical conductivity of 0.14 dS m^−1^, a pH of 6.7, and an organic matter content of 2.8%. The N, P, and K contents were 62, 87, and 412 kg ha^−1^, respectively. The pots were placed in two identical growth chambers (36 in each) and watered to FC. After 10 days, the seedlings formed were thinned to one uniformly growing seedling per pot. The two chambers (Percival model E-75L1; Percival Intellus Control Systems Ltd., Brisbane, Queensland) were modified to maintain different concentrations of the food-grade CO_2_ gas supplied from a G-size cylinder (Coregas Ltd., Brisbane, QLD), using an ADC 2000 CO_2_ monitor (ANRI Instruments and Controls Ltd., Melbourne, VIC), in conjunction with a solenoid valve, which together could modify the gas flow into the chamber. In all experiments, one chamber was set at a gaseous atmosphere [CO_2_] enriched to 700 ppm, whilst the second identical growth chamber was maintained at an ambient [CO_2_] of 400 ppm by daily flushing it with laboratory air. White light was supplied uniformly in both chambers (at pot level, *ca.* 800 µmol m^−2^ s^−1^) in a 12/12 h (day/night) photoperiod. Temperature and relative humidity were maintained at 30/20 ± 2 °C (day/night) and a constant 85% ± 5%, respectively, in both growth chambers.

### 4.2. Experimental Setup

The initial soil moisture level was determined and then adjusted to the required level using the procedure of Nguyen [43]. Briefly, *ca.* 10 kg of the soil to be used in the study was placed in three pots and saturated with tap water, the pot surface was covered with a black plastic sheet, and the pots allowed to drain for 48 h. After this time, the plastic sheet was removed, and three soil samples (each *ca.* 300 g) were taken from the mid-position of each pot. These wet soil samples were weighed (A) before being oven-dried at 90 °C for 72 h and then weighed again after drying (B).

Field capacity (*FC*) was calculated by the following Equation (1):

(1) FC (%)=(A−B)×100 ÷B

where *A* is the weight of wet soil samples and *B* is the oven-dried weight.

The soil moisture stresses studied included two treatments in which the amount of water applied to each pot at 2-day intervals was determined to keep soil moisture close to 100% and 50% of FC. Half of the pots (36) were randomly placed in one growth chamber (*ca.* 400 ± 50 ppm), while the remainder were placed into the other (*ca.* 700 ± 50 ppm). A repeated experiment was undertaken 15 days after the completion of the first experiment with the growth chambers reversed, with the ambient one being used for the elevated [CO_2_] and vice versa.

### 4.3. Glyphosate Application

When *S. cannabina* reached the four-leaf stage (*ca.* 21 days after planting), plants from both growth chambers were removed for the application of glyphosate (Weedmaster DST; 470 g L^−1^ glyphosate as potassium and mono-ammonium salt; Nufarm Australia). In Australia, the recommended rate of glyphosate for controlling all broadleaf and grassy weeds in glyphosate-resistant cotton is 1034 g ae ha^−1^ [44]. On this basis, the concentrations of glyphosate applied were (1) 0 g ha^−1^ (control), (2) 517 g ha^−1^ (50% of the recommended), and (3) 1034 g ha^−1^ (recommended rate). A herbicide track sprayer, set to deliver 200 L ha^−1^, was used for the application of the different concentrations of glyphosate. Application started with the lowest rate (517 g ae ha^−1^: 50% of recommended) and finished with the recommended dose (1034 g ae ha^−1^). After herbicide treatment, the plants were placed back in the growth chambers under the same conditions in which they were grown. The efficacy of glyphosate on *S. cannabina* was recorded as an injury score through visual observation after 21 days of spraying.

### 4.4. Data Collection

All data (viz. plant height, leaves per plant, growth index, root length, fresh biomass, dry biomass, leaf chlorophyll content, stomatal conductance, and weed control rating) were measured at harvest (21 days after spraying). At harvest (21 days after spraying), the shoot and root lengths of five randomly selected plants from one treatment were recorded with a measuring tape and then averaged to produce a single comparative estimate. Additionally, leaves per plant and whole-plant fresh and dry biomass (90 °C for 72 h) of five randomly selected plants from one treatment were recorded at harvest (21 days after spraying) and then averaged to produce a single comparative estimate. The growth index used is a quantitative indicator of plant growth rate and was calculated using the following Equation (2):

(2) $$Growth\;index\;\left(cm^{3}\right)=\pi \times {\left(\frac{w}{2}\right)}^{2}\times h$$

where *w* is the width of the plant calculated as an average of two widths (measured with a Vernier caliper), one measured at the widest point and the other at 90° to the first, and h is the plant height measured from soil surface to the last stem node at the top.

For chlorophyll measurements, a SPAD-502 Plus chlorophyll meter (Konica Minolta, Tokyo, Japan) was used to take readings from five fresh, fully expanded, healthy and lush green leaves from each plant after 21 days of spraying. The SPAD units were then averaged to produce a single comparative estimation of chlorophyll content. The stomatal conductance was measured between 10:00 am and 12:00 pm on five fresh healthy, fully expanded and undamaged penultimate leaves using an SC-1 Leaf Porometer (Decagon Device, Inc.) after 21 days of spraying and then averaged to produce a single comparative estimate of stomatal conductance. Data of the weed control rating to assess the crop damage were taken as per procedure [45] after 21 days of spraying. The experiment was conducted twice and involved swapping the growth chambers for [CO_2_] treatments. The data presented are the pooled data for both experiments.

### 4.5. Statistical Analyses

The experiment was arranged in a completely randomized design, giving equal importance to all treatment factors, with six replications per treatment. All the experiments were repeated. Data were subjected to ANOVA using IBM SPSS Statistics 25. Data from different experimental runs were combined because there was no significant treatment-by-experimental run interaction. Means were separated using Fisher’s protected LSD test at *p* = 0.05. Bar charts were prepared for all parameters using Sigma Plot v. 14.

## 5. Conclusions

Overall, 100% FC and an elevated [CO_2_] had a significant positive effect on the growth and biomass production of *S. cannabina*. This may be due to higher photosynthetic efficiency, lower stomatal conductance, and improved WUE of the plants grown in an elevated [CO_2_]. Soil moisture stress had a negative effect on the growth and biomass production of *S. cannabina* due to reduced physiology. Glyphosate application at the recommended rate resulted in the maximum reduction in weed biomass under all climatic conditions tested. However, glyphosate efficiency reduced to 63% with elevated [CO_2_] and moisture stress treatment, when applied at 50% of the recommended rate. The current study indicated the adaptability of *S. cannabina* to these climatic changes and difficulty in managing this emerging weed if plant age, operational conditions, and environmental conditions are not ideal for herbicide application and whenever a low effective dosage is delivered on this weed. Under elevated atmospheric [CO_2_] and moisture-limiting conditions, it is ideal to apply glyphosate at the recommended rate in order to ensure herbicide efficacy and effective control of *S. cannabina*.

## Figures and Tables

**Table 1:** Effect of different herbicide rates on weed control, as measured by the biomass reduction in S. cannabina under two different [CO_2_] and soil moisture conditions. The experiment was repeated, and the pooled data from the two studies are presented here.

Glyphosate Rate (g ae ha^−1^)	Percentage Control (Biomass Reduction)			
	400 ppm		700 ppm	
	100% FC	50% FC	100% FC	50% FC
517	100 a	94 a	100 a	63 b
1034 (recommended rate)	100 a	100 a	100 a	100 a
LSD (*p* ≤ 0.00) = 7.5				

Means with different letters in each column differ significantly at *p* < 0.05. Abbreviations: FC, field capacity; LSD, least significant difference.

**Table 2:** Effect of different herbicide rates on the weed control (injury score) of S. cannabina under different [CO_2_] and soil moisture conditions. The experiment was repeated, and the pooled data from the two studies are presented here.

Glyphosate Rate (g ae ha^−1^)	Percentage Injury Score			
	400 ppm		700 ppm	
	100% FC	50% FC	100% FC	50% FC
517	100 a	87 b	100 a	46 c
1034	100 a	100 a	100 a	100 a
LSD (*p* ≤ 0.00) =11.9				

Means with different letters in each column differ significantly at *p* < 0.05. Abbreviations: FC, field capacity; LSD, least significant difference.

## Data Availability

Not applicable.
